# Effects of phlorizin on diabetic retinopathy according to isobaric tags for relative and absolute quantification–based proteomics in db/db mice

**Published:** 2013-04-05

**Authors:** Shi-yang Zhang, Bao-ying Li, Xiao-li Li, Mei Cheng, Qian Cai, Fei Yu, Wei-dong Wang, Min Tan, Guang Yan, Shi-lian Hu, Hai-qing Gao

**Affiliations:** 1 Key Laboratory of Cardiovascular Proteomics of Shandong Province, Department of Geriatrics, Qi-Lu Hospital of Shandong University, Jinan, Shandong Province, China; 2 Department of Geriatrics, Anhui Provincial Hospital Affiliated to Anhui Medical University, Hefei, Anhui Province, China

## Abstract

**Purpose:**

Diabetic retinopathy (DR) is a leading cause of vision loss in working-age people. To retard the development and progression of retina lesions, effective therapeutic strategies directed toward key molecular targets are desired. Phlorizin is effective in treating diabetic complications, but little is known about functional protein changes that may mediate its actions. The aim of this study was to identify retinal proteomic alterations in db/db mice treated with phlorizin.

**Methods:**

We used C57BLKS/J db/db mice as a type 2 diabetic animal model, while C57BLKS/J db/m mice were selected as the control. Phlorizin (20 mg/kg bodyweight /d) was administrated to db/db mice for ten weeks. Serum fasting blood glucose and advanced glycation end products were determined. Meanwhile, retina cell apoptosis was determined with terminal transferase dUTP nick end labeling. Isobaric tags for relative and absolute quantification and subsequent liquid chromatography-tandem mass spectrometry (LC-MS/MS) were used to identify and profile retinal proteins among control, untreated diabetic, and phlorizin-treated db/db mice. The expression of glial fibrillary acidic protein was measured in retinas using western blotting analysis.

**Results:**

Phlorizin treatment significantly reduced fasting blood glucose and levels of advanced glycation end products (p<0.05) and remarkably inhibited retina cell apoptosis and the expression of glial fibrillary acidic protein in the retinas of db/db mice. In addition, we identified 1,636 proteins from retina tissue in total, of which 348 proteins were differentially expressed in db/db mice compared with the controls. Only 60 proteins in the retinas of the db/db mice were found to be differentially changed following phlorizin treatment, including 33 proteins that were downregulated and 27 proteins that were upregulated. Most of these differentially changed proteins were involved in oxidative stress, apoptosis, energy metabolism, and signaling transduction.

**Conclusions:**

Our study revealed the expression of proteins differentially changed after phlorizin therapy. These proteins are most likely to participate in the development and recovery of DR. Our findings help expand understanding of the mechanism underlying the onset and progression of DR, and provide novel targets for evaluating the effects of phlorizin therapy.

## Introduction

Diabetes is characterized by hyperglycemia, which contributes to macrovascular and microvascular damage. Diabetic retinopathy (DR) is a prevalent and profound complication of diabetes. Nearly all patients with type l diabetes and more than half with type 2 develop retinopathy [1]. Further, DR remains the leading cause of visual impairment and blindness among people of working age in the industrialized world [2]. Patients with DR are 25 times more likely to become blind than individuals without diabetes [1]. Thus, DR presents a tremendous health problem worldwide. However, current therapeutic options for treating DR, such as laser photocoagulation and intensive metabolic control, are limited by considerable side effects and are far from satisfactory; better strategies are required.

Numerous studies have demonstrated that oxidative stress plays a pivotal role in diabetic complications, including DR [3,4]. Reactive oxygen species has been implicated in contributing to the metabolic abnormalities in DR [5,6]. Administering antioxidants to diabetic rats could prevent the retina from undergoing oxidative damage and developing DR. Nevertheless, large-scale clinical trials with classic antioxidants have failed to demonstrate substantial beneficial effects on treating diabetic vascular complications [7,8]. Therefore, there is strong incentive to search for potential candidates that combat DR with few side effects. In addition, increased understanding of the mechanism by which the agents arrest the progression of DR is needed.

Phlorizin, a phloretin glucoside, is a dihydrochalcone and is mainly distributed in apple trees, where it acts as a natural antibacterial plant defense metabolite. Phlorizin has been reported to possess various properties, including being antioxidative, anti-inflammatory, anti-tumorigenic, and having the ability to lower plasma glucose concentrations and improve memory [9,10]. A series of studies were conducted using phlorizin to curb diabetic complications. In streptozotocin-induced diabetic rats, phlorizin prevented proteinuria, hyperfiltration, and kidney hypertrophy, alleviating early renal functional and preventing some structural changes in diabetes [11]. T-1095, a derivative of phlorizin, suppressed the development of albuminuria and the expansion of the glomerular mesangial area in db/db mice, indicating that the progression of diabetic nephropathy was prevented [12]. Previous studies also demonstrated that phlorizin attenuated high glucose-induced morphological and functional changes in cultured bovine retinal pericytes [13,14]. These studies suggest that phlorizin provides potential protection against diabetic microvascular complications, including DR, while little is discussed about the functional protein changes.

Our study was designed using db/db mice as an animal model to examine the effect of phlorizin on DR and to explore the mice retinal proteomes alterations occurring with diabetes and their responsiveness to phlorizin treatment using the iTRAQ approach. Here we identified differentially expressed proteins in the db/db mouse retina, of which some were back-modulated following treatment with phlorizin. These altered proteins might provide insight into the factors and mechanisms responsible for DR. These potential functional proteins might benefit early detection, help in monitoring the effects of DR therapy, and provide candidates for therapeutic targets.

## Methods

### Materials

Phlorizin (purity >98%, catalog no. 1,005,004–19) was provided by Jianfeng Inc. (Tianjin, China). Anti-mouse glial fibrillary acidic protein (GFAP) antibody was purchased from Proteintech Group Inc. (Chicago, IL). Anti-mouse glutaredoxin-3 (Glr×-3) antibody was purchased from Sigma-Aldrich Corp (St. Louis, MO). Anti-mouse γ-crystallin polyclonal antibody was purchased from Santa Cruz Biotechnology (Santa Cruz, CA). The terminal deoxynucleotidyl transferase biotin-dUTP nick end labeling (TUNEL) in situ apoptosis detection kit was purchased from R&D Systems (Minneapolis, MN). Eight-plex isobaric tags for relative and absolute quantification (iTRAQ) protein labeling kit/reagents were purchased from AB Sciex (Framingham, MA). All other reagents used were standard commercial high-purity materials.

### Experimental animals and treatment

Male C57BLKS/J [15] db/db and db/m mice (n=24, seven weeks old) were purchased from the Model Animal Research Center of Nanjing University (Jiangsu, China). They were housed in cages and received laboratory pellet chow and tap water ad libitum in a constant environment (room temperature 20–22 °C, room humidity 40%–60%) with a 12 h:12 h light-dark cycle. The mice were kept under observation for one week before the experiments started. All procedures were approved by the animal ethics committee of Shandong University. C57BLKS/J db/m mice were selected as the control group (CC, n=8). The db/db mice were divided into two groups: an untreated diabetic group (DM, n=8) administered normal saline solution by intragastric gavage and another diabetic group treated with a dosage of 20 mg/kg of phlorizin (DMT, n=8). Phlorizin was given with the same volume of normal saline solution by intragastric administration for ten weeks. Each group of mice was observed from week 7 to week 18 without any administration of hypoglycemic therapy. At the end of the intervention, all mice were fasted overnight and then euthanized by an overdose of carbon dioxide asphyxiation followed by cervical dislocation. Fasting blood were collected from the tail vein and stored in Eppendorf tubes at −80 °C. The eyes were immediately enucleated, and then the retinas were dissected. Retina tissue and sera were kept at −80 °C until further analysis.

### Estimation of bodyweight, blood glucose, and advanced glycation end products

The animals were weighed every week. Fasting blood glucose (FBG) was determined with the DVI-1650 Automatic Biochemistry and Analysis Instrument (Bayer, Leverkusen, Germany). Serum advanced glycation end products (AGEs) specific fluorescence determinations were performed by measuring emission at 440 nm on excitation at 370 nm using a fluorescence spectrophotometer (HITACHI F-2500, Tokyo, Japan).

### Terminal transferase dUTP nick end labeling

Whole eyes were fixed in 4% paraformaldehyde overnight at 4 °C and embedded in paraffin. The 5-μm-thick retina sections were then isolated and cut using standard histological procedures. The TUNEL assay was performed in tissue sections, as previously published [16,17] using the TUNEL kit. After washes with phosphate-bufferes saline (PBS; 137 mM NaCl, 2.7 mM KCl, 1.8 mM KH_2_PO_4_ and 10 mM Na_2_HPO_4_, PH7.4) twice, the retina sections were reacted with TUNEL reagents at 37 °C for 1 h and were then washed three times in PBS for 1 min at room temperature. After that, the sections were incubated with an antibody-peroxidase conjugate at room temperature for 30 min and were then developed using DAB tetrahydrochloride peroxidase substrate. In short, DNA fragments in the retinal sections were labeled with digoxigenin-nucleotide and then allowed to bind an anti-digoxygenin conjugated to a rhodamine reporter molecule. The number of TUNEL-positive cells per visual field was counted in a marked fashion and expressed as the percent of the total number of cells. A minimum of ten fields each in three groups was recorded per condition.

### Sample preparation and trypsin digestion for proteomic analysis

About 50 mg retina tissue from each of four mice per group was pooled and homogenized in the presence of liquid nitrogen, and then lysed with 500 μl dissolution buffer (4% sodium dedecyl sulfate, 100 mM dithiothreitol, 150 mM TrisHCl PH 8.0). After 5 min incubation in boiling water, the suspensions were sonicated using an ultrasonic cell crusher for 6 min (ten times, 80w, 10 s each time with 15 s intervals). Then the mixture was incubated at 100 °C for 5 min. The crude extract was clarified with centrifugation at 14000 g for 20 min. The filter-aided sample preparation (FASP) method allows gel-free processing of biologic samples solubilized with detergents for proteomic analysis with mass spectrometry. In FASP, detergents are removed with ultrafiltration, and after protein digestion, peptides are separated from undigested material. About 120 μg of proteins for each sample were incorporated in 30 μl dissolution buffer, incubated at boiling water for 5 min, cooled to room temperature, diluted with 200 μl UA buffer (8 M urea, 150 mM TrisHCl, PH 8.0) and transferred to 30 kDa ultrafiltration. The samples were centrifuged at 14,000 × *g* for 15 min, and 200 μl UA buffer was added. The samples were centrifuged for 15 min at the same conditions. Then 100 μl 0.05 M iodoacetamide in UA buffer was added, and the samples were incubated for 20 min in darkness. After 10 min centrifugation at the above conditions, the filters were washed three times with 100 μl UA buffer. Then 100 μl DS buffer (50 mM triethylammonium bicarbonate at PH 8.5) was added to the filters, and the samples were centrifuged for 10 min at the same conditions as before. This step was repeated twice. Finally, 2 μg trypsin (Promega, Fitchburg, WI) in 40 μl DS buffer was added to each filter. The samples were incubated overnight at 37 °C or 25 °C, respectively. The resulting peptides were collected with centrifugation. The filters were rinsed with 40 μl 10×DS buffer.

### Isobaric tags for relative and absolute quantification labeling and strong cation exchange separation

Concentration of the peptides can be estimated with an ultraviolet spectrometer (UV-2000, Unico, Shanghai, China) assuming that 0.1% solution of vertebrate proteins has at 280 nm an extinction of 1.1 absorbance units. About 60 μg peptides of each group were labeled with iTRAQ reagents (114 for the peptides of the C group, 116 for the peptides of the DMT group, and 117 for the peptides of the DM group) following the manufacturer’s instructions (Applied Biosystems, Foster City, CA).

The labeled samples were dried out and then diluted with 20 fold cation exchange binding buffer (10 mM KH_2_PO_4_ in 25% acetonitrile at pH 3.0). Strong cation exchange (SCX) chromatography was performed to separate the labeled samples into ten fractions with polysulfethyl A column (4.6×100 mm 5 µ, 200Å, PolyLC, Columbia, MD). A suitable gradient elution was applied to separate peptides at a flow rate of 1 ml/min with elution buffer (10 mM KH_2_PO_4_, 500 mM KCl in 25% acetonitrile at PH 3.0). Eluted peptides were collected and desalted with an offline fraction collector and C18 cartridges (Sigma, St. Louis, MO).

### Mass spectrometric analysis of isobaric tags for relative and absolute quantification samples

Mass spectrometric analysis was performed using a micro liquid chromatography system (MDLC, GE Healthcare, Little Chalfont, UK) and a LTQ-Velos ion trap mass spectrometer (ThermoFinnigan, San Jose, CA). The separation column was a 0.15 mm × 150 mm capillary packed with Zorbax 300SB-C18 particles (Agilent Technologies, Palo Alto, CA). The mobile phase A (0.1% formic acid in water) and the mobile phase B (0.1% formic acid in ACN) were selected. The volumetric flow rate in the separation column was set to about 1 μl/min, with a 2-h-long separation gradient running from 0% to 100% B.

Mass spectrometry (MS) data were acquired using data-dependent acquisition conditions: Each MS event was followed by zoom/MS2 scans on the five top-most intense peaks. Zoom scan width was ±5 m/z, and dynamic exclusion was enabled at repeat count 1, repeat duration 30 s, exclusion list size 200, exclusion duration 60 s, and exclusion mass width ±1.5 m/z. The pulsed-Q dissociation (PQD) parameters were set at isolation width 2 m/z, normalized collision energy 35%, activation Q 0.7, and activation time 0.1 ms. The threshold for MS/MS acquisition was set to 500 count.

### Data analysis

For protein identification and statistical validation, the acquired MS/MS spectra were automatically searched against the non-redundant International Protein Index (IPI) mouse protein database (version 3.72) using the Turbo SEQUEST program in the BioWorks 3.1 software suite. The database search parameters included the followings settings: the number of allowed missed tryptic cleavage sites was set to two, the peptide tolerance was 2 μ, the fragment ion tolerance was 1 μ, and only fully tryptic fragments were considered for peptide selection. The sensitivity threshold and mass tolerance for extracting the iTRAQ ratios were set to 1 and ±0.5, respectively. Data filtering parameters were chosen to generate false-positive protein identification rates of <1%, as calculated by searching the MS2 scans against a forward reversed database of proteins. The threshold was set to 1.5 with a p value <0.05 yielding at least a 50% change in abundance compared to the reference (the control group).

### Subcellular localization analysis and functional classification

The localization analysis of the identified proteins in retinas was performed by using AmiGO (Version 1.8). We got details including information about subcellular localization by manually inputting the protein names. The sequences for all proteins identified with iTRAQ were submitted to KOGnitor for KOG (eukaryotic orthologous groups) classification. When we manually inputted an identified protein sequence, it was assigned a KOG number. A KOG number belongs to one category. The protein ratio for each category was calculated by dividing the number of proteins within a category by the sum of the assigned proteins from all categories.

### Western blotting analysis for glial fibrillary acidic protein, γ-crystallin, and Glr×-3

Proteins were separated by electrophoresis in a SDS-polyacrylamide gel. After the proteins were transferred onto a polyvinylidene difluoride membrane, the blot was incubated with blocking buffer 1X phosphate-bufferes saline (PBS; 137 mM NaCl, 2.7 mM KCl, 1.8 mM KH_2_PO_4_ and 10 mM Na_2_HPO_4_, at PH7.4; and 5% non-fat dry milk) for 1 h at room temperature and then probed with primary antibodies: anti-mouse GFAP antibody (1:1000), anti-mouse γ-crystallin polyclonal antibody (1:1000), and anti-mouse Glr×-3 antibody (1:1000), followed by incubation with goat anti-rabbit conjugated with horseradish peroxidase-conjugated secondary antibody (New England Biolabs, Beverly, MA). To control equal loading of the total protein in all lanes, the blots were stained with antiactin antibody (1:2000; Sigma Aldrich, St Louis, MO). The intensities were quantified with densitometric analysis.

### Statistical analysis

Data are presented as mean±standard deviation. Statistical comparisons among the three experimental groups were made using the unpaired Student *t* test and one-way analysis of variance (ANOVA). A value of p<0.05 was considered statistically significant.

## Results

### Effects of phlorizin on bodyweights, fasting blood glucose, and advanced glycation end products

**Figure 1:**
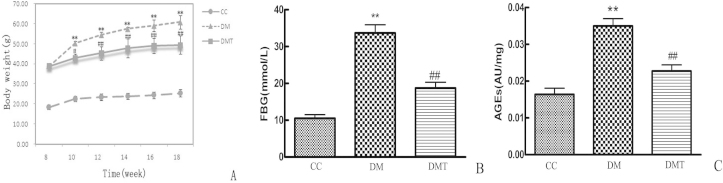
Effects of phlorizin on bodyweight, FBG, and AGEs in db/db mice. Comparisons of (**A**) bodyweight, (**B**) fasting blood glucose (FBG), and (**C**) advanced glycation end products (AGEs) among CC group, DM group and DMT group. **A**: The bodyweight of DM group was higher than that of CC group (**p<0.01), phlorizin significantly reduced bodyweight of db/db mice (##p<0.01). **B**: Treatment with phlorizin caused a elevated decrease in FBG (##p<0.01). **C**: Diabetes increased the level of AGES, while phlorizin treatment significantly reduced the diabetes-induced increase in AGES (##p<0.01). The abbreviations used are as follows: control db/m group (CC), untreated db/db group (DM), phlorizin treated db/db group (DMT). All data are expressed as the mean±standard deviation (SD; n=8/ each group, one way-ANOVA, Tukey test).

Figure 1 shows the results of the comparisons of bodyweights, FBG, and AGEs among these three groups. The bodyweight in the DM group was significantly higher than in the control group (p<0.01) during the entire experiment period. However, bodyweight was significantly inhibited at ten weeks, 12 weeks, 14 weeks, 16 weeks, and 18 weeks after phlorizin administration in the DMT group compared to the DM group (p<0.01; Figure 1A). The FBG and AGEs of the DM group were higher than those of the control mice (p<0.05). Moreover, the FBG and AGEs were significantly decreased by ten weeks of phlorizin administration in the DMT group when compared with the DM group (p<0.05; Figure 1B,C).

### Effect of phlorizin in retinal neurodegeneration (apoptosis and glial activation)

**Figure 2:**
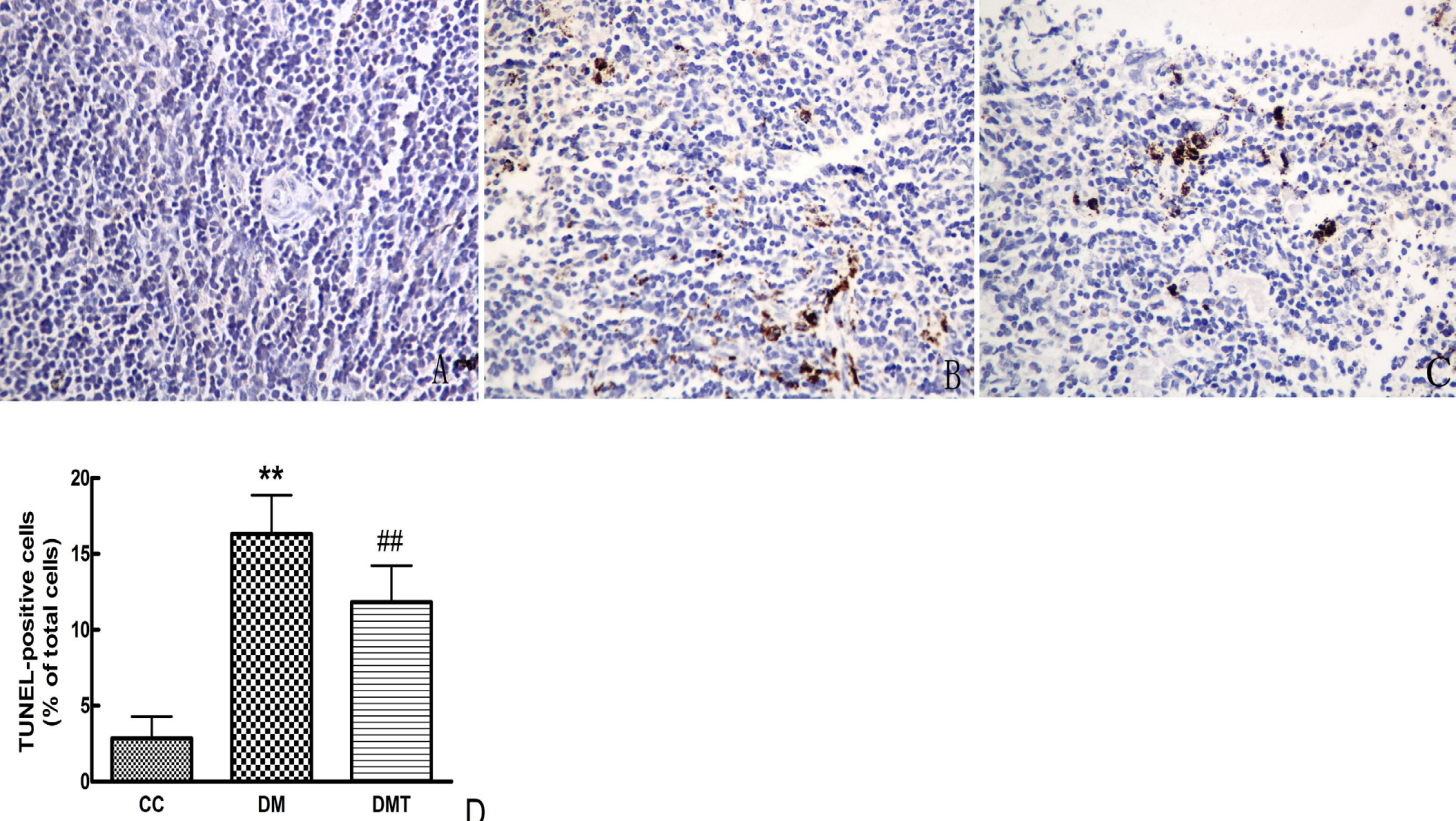
Effect of phlorizin on diabetes-induced retinal cells apoptosis with TUNEL assay (× 400). Terminal transferase dUTP nick end labeling (TUNEL) staing of retinal cells apoptosis in CC (**A**), DM (**B**), and DMT (**C**). **D**: The graph displayed the TUNEL-positive cells counted among three groups. Treatment with phlorizin significantly reduced retinal cells apoptosis as determind by TUNEL(##p<0.01). The abbreviations used are as follows: control db/m group (CC), untreated db/db group (DM), phlorizin treated db/db group (DMT). All data are expressed as the mean±standard deviation (SD; n=8/ each group, one way ANOVA, Tukey test).

As shown in Figure 2, TUNEL-positive cells developed nuclear staining. TUNEL staining was readily observed, and positive cells were predominantly located in the ganglion cell layer and the vascular endothelium. However, the mice in the control group showed little staining anywhere in the retina. In addition, when the db/db mice were treated with phlorizin, TUNEL staining was attenuated. In contrast, the number of TUNEL-positive cells in the DM group increased significantly compared to the control group (p<0.01). Treating db/db mice with phlorizin significantly reduced the number of TUNEL-positive cells (p<0.01).

**Figure 3:**
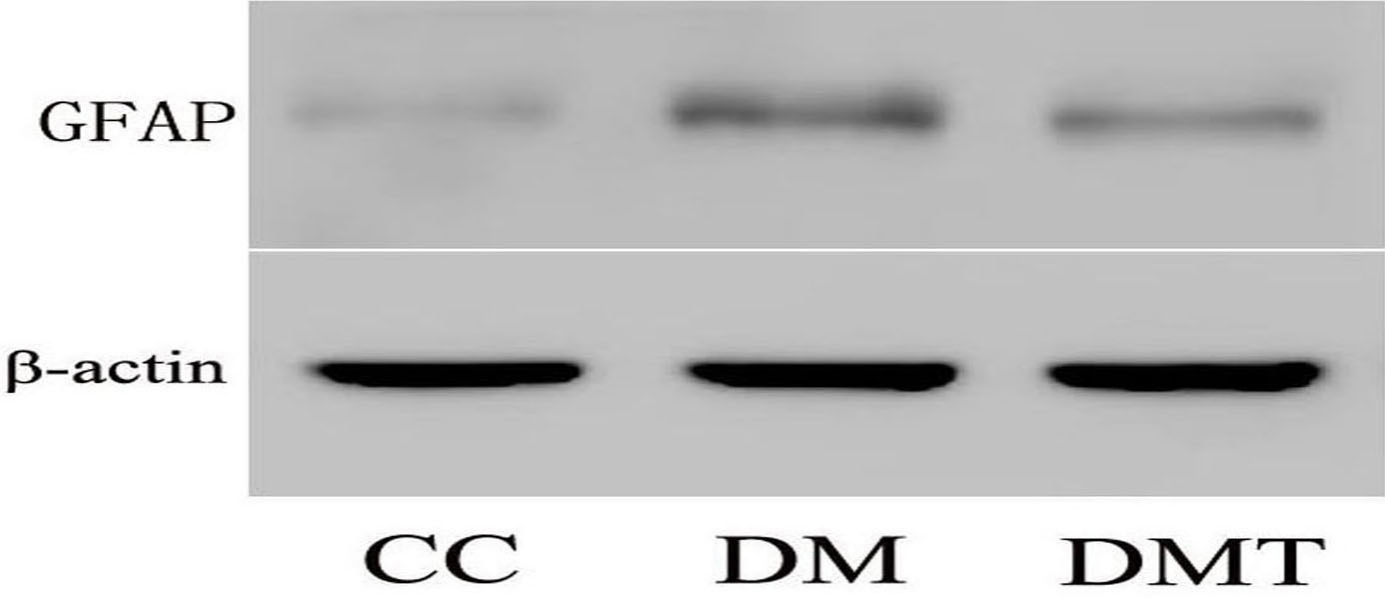
Effect of phlorizin on glial fibrillary acidic protein expression by western blotting. β-actin was used as the loading control. CC: control db/m group; DM: untreated db/db group; DMT: phlorizin treated db/db group.

Neuroglial activation was demonstrated in db/db retinas by an increase in GFAP expression compared with the control group. In contrast, phlorizin treatment downregulated the retinal GFAP expression in db/db mice (Figure 3).

### Isobaric tags for relative and absolute quantification proteomics profiling on the effect of phlorizin in the db/db mice retina

Protein profiling was analyzed using the iTRAQ approach. A total of 1,636 proteins were identified. A strict cutoff value of a 1.5-fold change was used for identifying differential proteins. The false-positive rate was set at <1% to guarantee the accuracy of the results. Among which 348 proteins were differentially expressed in the diabetic retina in comparison to the control, comprised of 177 proteins that were increased and 171 proteins that were decreased. Moreover, to examine the effect of phlorizin on the proteome change, proteome analysis was also conducted on the phlorizin-treated diabetic retinas. Of the significantly changed proteins between the DMT group and the DM group, 33 proteins were downregulated with the treatment of phlorizin, while 27 proteins upregulated, as shown in the appendix (Appendix 1). Briefly, the proteins that back-regulated following phlorizin treatment were involved in various aspects of important biologic functions, including metabolism, oxidative stress, structure activity signaling transduction, cell proliferation and growth, apoptosis, and inflammation response.

### Subcellular localization analysis and bioinformatic functional analysis phlorizin associated retina proteins in db/db mice

**Figure 4:**
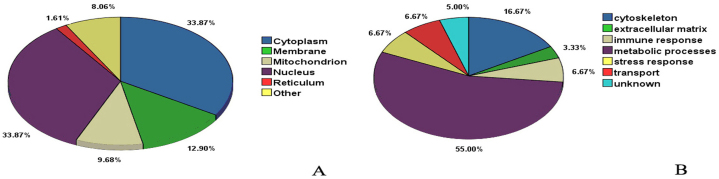
Subcellular localization and functional classification analysis of the identified differential proteins between untreated db/db mice and phlorizin treated db/db mice. A, Subcellular localization by AmiGO analysis of the identified differential proteins between untreated db/db mice and phlorizin treated db/db mice. **B**: Functional classification by KOG analysis of the identified differential proteins between untreated db/db mice and phlorizin treated db/db mice.

The localization analysis of the identified proteins in retinas using AmiGO (Version 1.8) is shown in Figure 4A. Among these proteins, some are located in one or more position of the cell, 33.87% were in the cytoplasm, 33.87% in the nucleus, 12.90% in the plasma membrane, 9.68% in mitochondria, and 1.61% in the endoplasmic reticulum. The functional classification of the identified proteins in the retinas is shown in Figure 4B. Among the functional assignment of the proteins, 55.00% were in metabolic processes, 16.67% in the cytoskeleton, 6.67% in the stress response, 6.67% in the immune response, 6.67% in transport, and 3.33% in the extracellular matrix.

### Effect of phlorizin on γ-crystallin and Glr×-3 expression with western blotting

**Figure 5:**
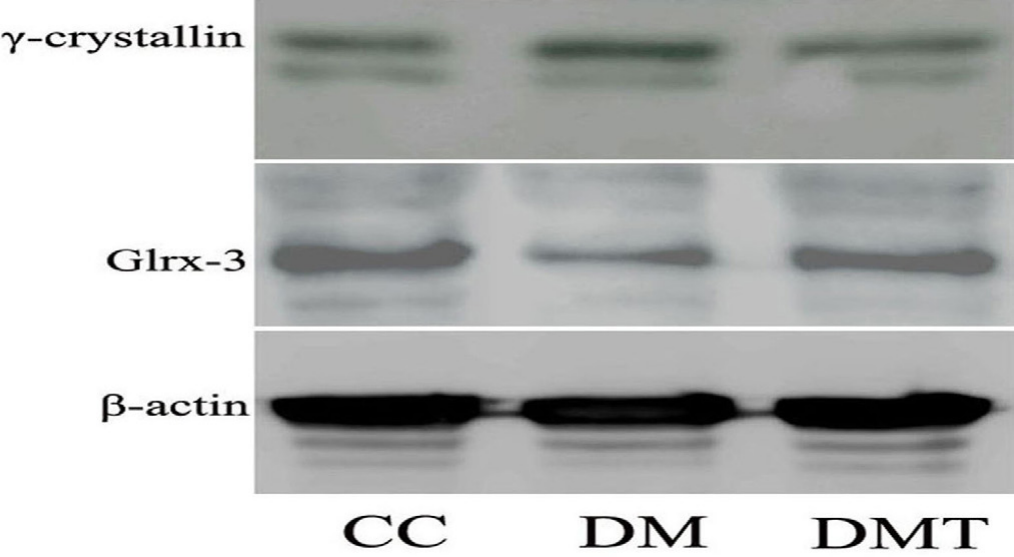
Effect of phlorizin on γ-crystallin and Glr×-3 expression by western blotting. β-actin was used as the loading control. CC: control db/m group; DM: untreated db/db group; DMT: phlorizin treated db/db group.

To provide confirmation of differentially expressed proteins, two candidate proteins were validated using western blotting analysis. γ-crystallin was inhibited whereas Glr×-3 was enhanced in the DMT group compared to the DM group (Figure 5). This result verified the reliability of the iTRAQ results.

## Discussion

As the most well known ocular complication of diabetes, DR is reaching epidemic proportions and becoming a debilitating public issue around the world [18]. This problem is aggravated due to the increased risk of all-cause mortality and cardiovascular events in patients with diabetes accompanying the prevalence of DR [19]. Thus, DR presents a frightening prospect to patients and frustrates physicians. Good glycemic control and laser photocoagulation remain the best standards of care for DR over decades, but neither one is regarded as optimal because they have limitations. Thus, there clearly is incentive to review the full range of metabolic dysregulation that contributes to DR to provide new therapeutic tools.

Phlorizin is a natural product and dietary constituent primarily present in several fruit trees, and is especially abundant in apple peels. Phlorizin makes up a large proportion of flavonoids manufactured by all plant families. Many studies have suggested that phlorizin displays potent antioxidant activity in peroxynitrite scavenging and inhibiting lipid peroxidation [20-22]. Our results indicated that the db/db mice showed higher AGEs relative to their counterparts, while the db/db mice administered phlorizin showed decreased AGEs levels. Chronic hyperglycemia favors glycation reactions and nonenzymatic glycation that can lead to interactions with amino acids in proteins, lipids, and nucleic acids to form AGEs [23]. Moreover, the accumulation of AGEs has been documented that interacted with oxidative stress. Therefore, we think that phlorizin’s antioxidant ability has a correlation with AGE reduction. In the present study, phlorizin treatment remarkably reduced serum glucose levels in db/db mice from the initial value. We also found a concomitant bodyweight loss in db/db mice with phlorizin treatment. Phlorizin, as a sodium-glucose cotransporter inhibitor, has the potential to promote weight reduction, due to the loss of glucose in the urine. The veterinary literature has suggested that chronic administration of phlorizin in lactating cows induces lipolysis [24], and dapagliflozin, a phlorizin analog, induces reduced adiposity, thus perhaps accounting for some weight loss.

Recently, findings have emerged that strongly support the idea that retinal neurodegeneration is an early event in the pathogenesis of DR that may predate and participate in the microcirculatory abnormalities that occur in DR [25,26]. Neuroretinal degeneration could activate metabolic and signaling pathways involved in the microangiopathic process, as well as in the disruption of the blood–retinal barrier, a crucial element in the pathogenesis of DR. In this light, it is reasonable to hypothesize that novel intervention based on neuroprotection will be effective in preventing and arresting DR development. In the current study, we have evaluated the effect of phlorizin in retinal neurodegeneration associated with diabetes using db/db mice, the model that best reproduces the neurodegenerative features observed in patients with DR. We found elevated amounts of TUNEL-positive cells in diabetic versus nondiabetic retinas, confirming the increased incidence of apoptosis, and we noted that this apoptotic activity was located in the endothelial, pericyte, and ganglion cell layers. Our results correlate with others, who also reported the death of retinal neural cells occurred during the course of diabetes, especially in the early stage [27,28]. Of note, in our study, treatment with phlorizin reduced diabetes-induced retinal cell apoptosis, as detected with the TUNEL array. Moreover, we have shown the upregulation of GFAP, which is generally considered the key feature of gliosis and a hallmark of glial cell activation [29], from the retinas of db/db mice. Our observation is consistent with previous reports that showed GFAP induction in db/db mice [30]. In addition, the present study provides evidence that the diabetic-induced glial response in the retina and the expression of GFAP decreased after phlorizin was administered. Taken together, these results suggest that phlorizin plays a crucial role in preventing neurodegeneration in db/db mice. Thus, phlorizin could be of potential benefit in preventing diabetic retinal damage and is a promising therapeutic agent for DR.

In this study, with the help of iTRAQ technology, we performed a comprehensive proteomics analysis of the db/db mice retina under the diabetes state and with phlorizin treatment. Using this approach, a total of 348 proteins were identified as differentially expressed in the db/db mouse retina with high confidence; among the changed proteins of the db/db mice, 60 proteins were back-regulated after phlorizin therapy. The back-regulated proteins were concomitant with the recovered AGEs as well as the improvement of DR pathological changes, including inhibition of diabetic apoptosis and neuronal cell injury. To the best of our knowledge, this is the first report regarding retina proteome alterations in db/db mice before and after phlorizin treatment.

The results from our proteomic study show that γ-crystallin was upregulated in retinas from db/db mice by at least fourfold and was back-regulated following phlorizin treatment. γ-crystallin along with α-crystallin and β-crystallin make up the three major families of crystallins. Crystallins, initially described as lens-specific structural proteins, now are thought to be multifunctional proteins with physiologic roles in non-lens tissues as well [31]. Our previous work and other groups revealed that α-crystallin isoforms were induced in the retinas of diabetic rats [32,33]. A recent study demonstrated that γ-crystallin, together with β-crystallins, may be involved in mediating vascular stabilization, remodeling, or survival in the developing mammalian eye, which is fundamental to normal ocular development and to the pathogenesis of numerous diseases, especially DR [34]. A novel finding here was that phlorizin treatment partly reversed the upregulation of γ-crystallin subjected to diabetes. Therefore, the modulatory effect of phlorizin on γ-crystallin might at least partly contribute to improving DR.

Importantly, Glr×-3 was found downregulated in the retinas of db/db mice and back-regulated to normal after phlorizin therapy. Glrx, also known as thioltransferase, serves as a general disulfide reductase for maintaining and regulating the cellular redox state and redox-dependent signaling pathways transduction by catalyzing reversible protein S-glutathionylation [35]. Given the general importance of these processes, Glrx has played a pivotal role in various disease-related conditions, including ischemic heart disease, cardiomyopathy, atherosclerosis, diabetic retinopathy, brain ischemia, and pulmonary diseases [36]. Knowledge regarding the role of Glrx as a regulator of apoptosis in mammalian cells, notably cardiomyocytes, has increased substantially. Moreover, the different isoform of Glrx in the experiment conditions may be attributed to the expression discrepancy between their data and ours. In detail, four different Glrx have been identified in mammalian cells, including Glr×-1, Glr×-2, monothiol Glr×-3 (PICOT), and Glr×-5. Generally, Glr×-1, the most well characterized protein in the Glrx family, mainly reside in cytoplasm. Glr×-3, expressed in our work, is called PICOT [37]. Human Glr×-3 is a multidomain monothiol Glrx and a homolog of yeast’s Glr×-3 and Glr×-4. Glr×-3/PICOT was first identified in a two-hybrid screen aiming at identifying protein kinase C (PKC)–interacting proteins [38]. Further, Glr×-3 was verified as a direct target of serum response factor in p19 cardiac cell differentiation, implying a role for this monothiol Glrx in the early embryonic development of cardiac tissue [35]. Jeong et al. have documented that Glr×-3/PICOT, as a putative PKC inhibitor, inhibited cardiac hypertrophy and enhanced ventricular function and cardiomyocyte contractility [39]. These studies have shown the relationship between Glr×-3 and cardiac hypertrophy; nevertheless, the role of Glr×-3 in the DR is still elusive. This is the first report of underexpression of Glr×-3 in the retina induced by the diabetes state. Importantly, the protein Glr×-3 change was almost normalized following phlorizin treatment, indicating Glr×-3 could ameliorate the development of DR.

Choosing several proteins that better elucidate the expression of changing proteins regulated by phlorizin is reasonable. As addressed above, the two candidate proteins were validated using western blotting analysis. γ-crystallin was inhibited whereas Glr×-3 was enhanced following phlorizin treatment, which verified the reliability of the iTRAQ results. Our previous work and other reports observed the expression of α-crystallin isoforms in the retina in a disease state such as diabetes [32,33], so it may be more interesting to explore the role of γ-crystallin isoform in the retina occurring with diabetes and related treatment. Moreover, other studies have shown that Glr×-3 belongs to the thiol transferase superfamily, which plays a crucial role in regulating redox and protecting cells against apoptosis as well defending as against reactive oxygen species [36]. Thus, further research regarding the link Glr×-3 with the diabetic retinopathy is required.

In conclusion, the present study reported that altered proteins in db/db mice completely returned to control levels or partially normalized, accompanying AGE recovery and retinal lesion improvement. These findings strongly support that back-modulated proteins, such as γ-crystallin and Glrx, might be involved with the development and improvement of DR. Reversible proteins were mainly linked to oxidative stress, apoptosis, signal transduction, energy metabolism, and inflammation regulation. Therefore, phlorizin treatment could deliver significant benefit to DR mainly by regulating the processes mentioned above. The proteins involved might form the basis of functional regulation. Further validation is required before they can be used as the first stages toward developing a panel of protein biomarkers or treatment targets for DR therapy evaluation. Moreover, of particular interest for future studies are the proteins not reversed by phlorizin treatment. Therefore, whether those unchanged proteins are linked to DR pathogenesis demands further investigation.

To the best of our knowledge, we have provided a proteomic inventory of db/db mice before and after phlorizin treatment for the first time, and disclosed some alterations in proteins back-regulated following phlorizin therapy. These proteins may play a crucial role in deterioration and restoration as important functional proteins, and provide insight into novel possible preventative and therapeutic targets of DR.
